# Array Biosensor for Toxin Detection: Continued Advances

**DOI:** 10.3390/s8128361

**Published:** 2008-12-15

**Authors:** Chris Rowe Taitt, Lisa C. Shriver-Lake, Miriam M. Ngundi, Frances S. Ligler

**Affiliations:** 1 Center for Bio/Molecular Science & Engineering, Naval Research Laboratory, Code 6900, Washington, DC 20375-5348, USA; E-Mails: lisa.shriverlake@nrl.navy.mil (L. C. S.); frances.ligler@nrl.navy.mil (F. S. L.); 2 Food and Drug Administration, N29 RM418 HFM-434, 8800 Rockville Pike, Bethesda, MD 20892, USA; E-Mail: Miriam.Ngundi@fda.hhs.gov (M. M. N.)

**Keywords:** toxin, detection, biosensor, multi-analyte, multiplex, food, clinical diagnostics

## Abstract

The following review focuses on progress made in the last five years with the NRL Array Biosensor, a portable instrument for rapid and simultaneous detection of multiple targets. Since 2003, the Array Biosensor has been automated and miniaturized for operation at the point-of-use. The Array Biosensor has also been used to demonstrate (1) quantitative immunoassays against an expanded number of toxins and toxin indicators in food and clinical fluids, and (2) the efficacy of semi-selective molecules as alternative recognition moieties. Blind trials, with unknown samples in a variety of matrices, have demonstrated the versatility, sensitivity, and reliability of the automated system.

## 1. Introduction

**Figure 1.**
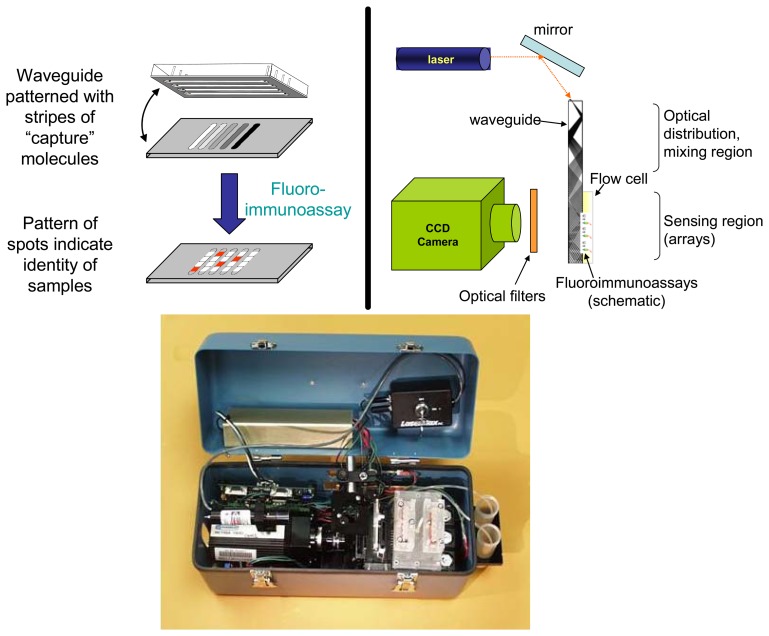
The NRL Array Biosensor. Upper left: Physically-isolated patterning and sample analysis leads to formation of an array of fluorescent spots on the waveguide surface. Upper right: Optical configuration of the light source, waveguide, and CCD camera. Lower panel: Automated prototype of the NRL Array Biosensor. Imaging components of the system are found on the left side of the box, whereas components for fluid handling are found on the right side of the box. Segregation of the fluidics from the expensive electronic components ensures that damage from any potential leakage is minimal. The entire system weighs less than 7 kg.

The NRL Array Biosensor is an optical biosensor system designed especially for simultaneous detection of multiple targets in multiple samples. In this system, antibodies or other “capture” molecules are immobilized in a two-dimensional array on an optical waveguide (as either stripes or spots) and standard fluoroimmunoassays are performed within the channels of a multi-channel flow cell placed on the waveguide surface (Figure 1, upper left). The fluorescence-based assays are then interrogated using evanescent wave technology: light from a 635 nm diode laser is focused into the edge of the patterned slide/waveguide and after propagation and mixing within the waveguide, the confined beam produces an evanescent field within the sensing portion of the waveguide. Surface-bound molecules labeled with fluorophore are excited by this evanescent field, producing a fluorescence signal; this fluorescence is then detected using a CCD camera fitted with appropriate bandpass and longpass filters (Figure 1, upper right). Since the penetration depth of the evanescent field is limited, only surface-bound fluorophores are excited, enabling analysis of non-homogeneous or turbid samples. The locations and intensities of the fluorescent spots indicate the identity and concentration of the target sample in each lane.

In 2003, we published a review summarizing the documentation of the use of the Array Biosensor for the detection of toxins [1]. The review documented that the Array Biosensor could: (1) test for multiple toxins simultaneously in multiple samples, (2) detect toxin levels as low as 500 pg/mL, (3) quantify the toxin concentration, and (4) perform toxin assays in clinical, food, and environmental samples. Both sandwich immunoassays for protein toxins (e.g., staphylococcal enterotoxin B [SEB] and ricin) and competitive immunoassays for low molecular weight toxins (e.g., trinitrotoluene and fumonisin B1) were reported. In this review, we describe the progress in instrument development and toxin detection over the five years since the prior review. The Array Biosensor has been automated and miniaturized for operation at the point-of-use, quantitative immunoassay arrays have been demonstrated against an expanded number of toxins and toxin indicators in food and clinical fluids, and semi-selective recognition molecules have been used to expand the repertoire of toxins that can be detected on a single array.

The Array Biosensor used in literature reports prior to 2003 was not an automated system. Assays were performed by pumping the samples and fluorescent reagents over arrays of capture molecules immobilized on a waveguide using flow channels molded into blocks of polydimethylsiloxane (PDMS). After a final wash, the processed array was dried and, on a separate system, illuminated using evanescent excitation light and imaged using a Peltier-cooled CCD camera. Semi-automated software was used to quantify the fluorescence in spots modified with capture antibodies. The flow cells required removal from the waveguide prior to imaging because the PDMS flow channels would scatter or absorb the evanescent illumination, weakening the signals and increasing the background. The introduction of a reflective layer between the waveguide and the flow cell prevented these two effects, so that the flow channels could remain in place during the imaging step [2]. The invention of the reflective layer was the key to automating the Array Biosensor. In the automated NRL system (Figure 1, lower panel) [3], all the operator has to do is fill six reservoirs with the sample to be tested and six more with the fluorescent reagents, insert the waveguide, and press “run” on the pre-programmed computer. While not quite as small as the NRL Array Biosensor, a commercial version of the fully automated device is available [4] with accessories specific for isolating pathogens from large volumes of water from food washes (www.hansontechnologies.com). Data described in this paper were taken with either the manual or automated system; results are comparable in terms of sensitivity of detection. We continue to use the manual system for assay development simply because the automated system was arbitrarily built to handle six samples simultaneously, whereas the manual system can handle as many as 12 samples simultaneously.

There is a clear need for measuring toxin levels for food safety, military, and Homeland Security applications. Toxins occur naturally in the food supply. Particularly in wet climates, mycotoxins produced by fungi can contaminate grain crops, threatening both livestock and people. Furthermore, mycotoxins can persist even after rigorous food processing [5] and may act as carcinogens. Better known are food poisonings caused by toxins, such as *Staphylococcus aureus* enterotoxins and botulinum toxins, which have been secreted into contaminated foodstuffs by bacterial growth. Toxic pollutants such as pesticides, chemical wastes, and explosives can contaminate the environment or be ingested with food or drinking water. Finally, bioterrorism raises the specter of exposure to toxins that can be weaponized, such as ricin, botulinum toxin, SEB, mycotoxins, and saxitoxin. Not only do we need rapid, sensitive methods for detecting all of these classes of toxins in food, water, and air samples, but we also need methods for monitoring for clinical exposure. It is desirable to have the ability to detect toxins directly in clinical fluids, but since many of the toxins are cleared rapidly from the circulation, detection of circulating anti-toxin antibodies provides an alternative approach to diagnose toxin exposure.

In addressing the requirements for toxin detection in such a wide variety of situations, we have designed the NRL Array Biosensor to meet the following requirements: 1) Minimal if any sample preparation is used. Solid food or environmental samples are simply homogenized and either filtered or centrifuged. Fluid samples may be diluted with a buffer to control pH or to reduce viscosity, but no preconcentration or fractionation of the sample is performed. 2) The assay time is adapted for the application. In many cases, rapid responses are more important than maximum sensitivity, and 10-15 minute assays are the norm. However, the sensitivity is proportional to assay time, so that increased sensitivity can be obtained if the exposure of the sample to the capture array is extended. For assays where sensitivity is more important than assay speed, 30-60 minute assays are conducted. 3) All assays are geared for operation by users without a technical background. We have demonstrated that assays for detection of toxin in food and environmental samples can be performed by users with as little as two hours of training on the automated biosensor. 4) The specificity of the assays is adapted to user needs. For a high degree of selectivity, immunoassays are the method of choice. However, they do require that the user have previous knowledge of what toxins might be present. We also demonstrate the detection of toxins using semi-selective molecules, such as sugars and antimicrobial peptides, which can recognize families of toxins. Such a strategy significantly expands the number of toxins that can be detected in a single test.

## 2. Applications for food, environmental testing

In the past several years, contamination of food whether accidental or deliberate has been an issue of concern throughout the world. Food contamination, poses both health risks and devastating economic vulnerability. Though most foodborne contamination is from bacteria, toxins also play a prominent role. Toxins are produced by organisms such as bacteria, fungi, and plants, and range in size from a few hundred daltons to large proteins in excess of several hundred kilodaltons; man-made toxins such as pesticides will not be discussed here. The amount of toxin required to cause harm varies from toxin to toxin: 1 ng/kg of botulinum toxin is deadly, whereas a similar dose of SEB would cause minor problems. Many of these toxins are associated with food-borne illnesses.

There are a variety of methods to detect biologicallyderived toxins including high pressure liquid chromatography, mass spectrometry, and Enzyme-Linked ImmunoSorbent Assays (ELISA). These procedures are time consuming, labor intensive, costly, and usually test for one compound at a time. In most cases, significant sample preparation must be performed to eliminate interference from components in the sample matrix. The NRL Array Biosensor was developed to detect different types of compounds simultaneously in real time with little, if any, sample preparation.

### 2.1. Large Protein Toxins in Foods

Detection of SEB in clinical, environmental, and food samples with the NRL Array Biosensor has been discussed in detail [1, 6]. With large protein toxins, a sandwich immunoassay format is used and has generally involved patterning biotinylated capture antibodies across the short axis of a NeutrAvidin-treated slide. After patterning, the slide is blocked to prevent non-specific adsorption, dried, and stored at 4°C or room temperature until use. For most assays, antibody-coated slides are exposed to samples for 8 to 15 minutes depending on the antigen and the sensitivity required. After exposure to the sample, a fluorescently-labeled tracer antibody was flowed over the slide for 4-8 minutes, rinsed with buffer two times, and then imaged. The identity and estimated concentration were determined by the location and intensity of the fluorescence spot. A full description of the analysis can be found in Ligler *et al.* [1]. In the automated system, up to 6 samples can be analyzed simultaneously while the non-automated system can test up to 12 samples.

**Figure 2.**
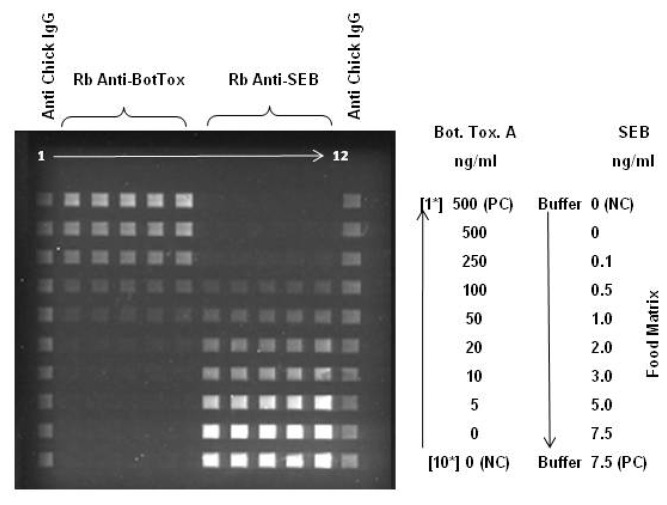
Simultaneous detection of botulinum toxoid A and SEB spiked into apple juice. The slide was patterned with anti-botulinum toxin (left half) and anti-SEB antibodies (right half). Apple juice samples spiked with botulinum toxoid A (concentration decreasing) and SEB (concentrations increasing) were flowed over the patterned surface. Subsequent incubation with a mixture of labeled “tracer” antibodies directed against both toxins was followed by imaging.

A detection limit of 0.1 ng/mL SEB has been achieved in several food matrices in less than 30 minutes including minimal sample processing and analysis [1, 6, 7]. Since the last review, these studies were expanded to include inactivated botulinum toxin A. Simultaneous detection of SEB and botulinum toxoid A (BotA) was demonstrated in various food matrices with the NRL Array Biosensor with little loss in sensitivity for either SEB or BotA for most matrices [3, 7]. Figure 2 shows the detection of SEB and BotA in buffered apple juice. Cholera and ricin have also been detected with the Array Sensor with detection limits as low as 1.6 and 8 ng/mL, respectively [1].

### 2.2. Mycotoxins in foods and air

Array Biosensor assays for bacteria and large toxins employ a sandwich immunoassay format. However, mycotoxins are smaller in size and are therefore better assayed using an indirect competitive immunoassay. We have demonstrated the NRL Array Biosensor for the detection of mycotoxins (ochratoxin A, deoxynivalenol and aflatoxin B_1_) in various food matrices and in air [8-11]. The Array Biosensor was used for detection of the mycotoxins individually and in combinations using both versions of the Biosensor.

Briefly, the competitive assay protocol involves attaching the biotinylated mycotoxin derivatives onto a waveguide, incubating the test sample with cyanine 5 (Cy5)-labeled anti-toxin antibodies and passing the pre-incubated mix over the immobilized mycotoxin derivatives. The immobilized mycotoxin derivatives compete with the toxin in the test sample for binding to the fluorescent antibodies. Therefore, the fluorescent signal resulting from the immunocomplex on the waveguide surface is inversely proportional to the concentration of toxin in the sample (decrease in signal with increasing concentration). The biotinylation procedure for each mycotoxin is dependent on its chemical nature. However, the immobilization of the biotin-mycotoxin conjugate, preparation of the Cy5-labeled antibodies, introduction of reagents onto the waveguide, fluorescent imaging, and data acquisition and analysis are similar to those employed for sandwich assays [3, 12].

In all cases, the initial step in developing the competitive assay has involved a checkerboard assay for each individual mycotoxin to determine the optimal concentrations of both the biotinylated derivative and the Cy5-labeled antibodies used in the assay individually and in combination. Competitive assays for the mycotoxins in buffer and spiked into various foods are then performed using the optimized conditions. Solid foods with coarse texture (barley, wheat pasta, oats, and cornflakes) are blended to a fine texture, while finer textured foods (cornmeal, roasted coffee) can be used as purchased. After spiking, the mycotoxins were extracted using a simple methanol/water mix, followed by a quick centrifugation. Wine samples were treated using three different protocols to minimize the effect of polyphenols in the matrix.

Prior to analysis, samples were diluted in buffer containing Cy5-labeled and incubated for 10-20 minutes before passing over the immobilized biotin-mycotoxin conjugates. Analysis of the mycotoxins using the manual Array Biosensor has demonstrated detection limits similar to those reported in literature [8, 9]. However, the sensitivity was slightly decreased when the automated version was employed for the analysis. This increase was attributed to differences in the optical configuration as well as slight differences in the assay protocol and fluid movement.

To determine whether the NRL Array Biosensor could be used to detect aerosolized toxins, deoxynivalenol - used as a model toxin – was spiked into aqueous extracts of air samples taken in the lab and analyzed using the system. Although not demonstrated in samples air taken from contaminated buildings, the low detection limit (4 ng/mL), combined with the rapid rate of air sampling during the study, demonstrated that the Array Biosensor is sufficiently sensitive to detect levels of deoxynivalenol encountered in dust and air samples found in agricultural processing facilities [9].

### 2.3 Blind Laboratory Trials

The methodology to determine detection limits is statistically-based; the detection limit has been defined as the lowest concentration tested yielding a net signal higher than 3 standard deviations of the negative controls. However, the true test of an instrument's sensitivity and reliability is a blind trial. Such trials can be designed to determine the usable detection limit (especially important when the presence of target is not known), the true time-to-result, time-to-failure (if any), user-friendliness, and false-positive and false-negative rates. Data obtained from such trials are useful in assessing the maturity of a given technology and its readiness for transition to use outside the laboratory.

**Figure 3.**
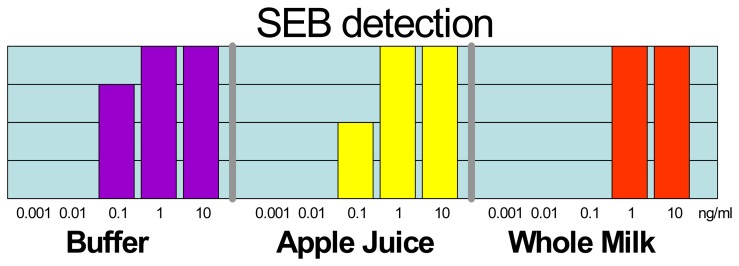
Results from blind trial of foods spiked with toxin. Shown is the number of samples (out of four replicates) designated “positive” by the Array Biosensor at each concentration tested in the different matrices.

To demonstrate the utility of the automated prototype of the NRL Array Biosensor for the detection of bacteria and toxins in foods, a blind laboratory trial was conducted with the assistance of the U.S. Food and Drug Administration (FDA) [13]. The FDA prepared 216 samples containing buffer (controls), SEB (1 – 10,000 pg/mL), *Salmonella typhimurium* (5 × 10^3^ – 5 × 10^7^ colony-forming units/mL), or an unnumbered strain of *Campylobacter jejuni* (used as another control) in three food matrices: water, apple juice, and milk; at least four replicates of each target at each concentration (in each matrix) were provided. The automated assay was completed in 45 minutes (sample preparation to analyzed assay) with the data analyzed visually by the operator or using a computer analysis program developed at NRL. Sample preparation steps consisted of neutralization with NaOH and the addition of 1/10 volume of 10 × buffer. For each sample, a set of positive and negative controls was run in parallel. All samples spiked with SEB at 1 ng/mL or higher were detected as positive with the exception of a 1 ng/mL milk sample (Figure 3); milk is commonly considered to be a troublesome matrix. Half of the SEB samples in water and apple juice were correctly identified at concentrations as low as 0.1 ng/mL. The false positive rate was approximately 1.7% by visual determination and 7.1% by computer evaluation; samples spiked with *C. jejuni* were responsible for half the false-positives. Full results of the laboratory trial can be found in Shriver-Lake *et al.* [13]

In a separate blind field demonstration, the NRL Array Biosensor was set up in a trailer at Dugway Proving Grounds, Utah to detect inactivated biological warfare agents in water [14]. The antibodies used as capture and tracer species were designated by the sponsor and were not the most sensitive combinations. All aspects of the analysis process were evaluated, including sample preparation time, assay time, data analysis time, waste production, ease of use, specificity and sensitivity. Although most of the biological agents tested were bacteria, a subset of the blind samples was spiked with botulinum toxoid as a representative toxin. A total of 316 blind samples were analyzed in a two week period, averaging 30-32 samples per eight hour day plus an additional two controls for every slide. Eighty-five percent of the botulinum samples with 100 ng/mL toxoid were correctly identified.

Two operational aspects of the NRL Array Biosensor were clearly demonstrated in this latter field trial. One of the operators had limited training (<2 hours) prior to running assays for a week, which demonstrated the ease of use of the system. Another aspect of the system was the generation of waste which is considered hazardous when testing for hazardous material. The waste generated from the NRL Array Biosensor was low when compared to other analytical systems - <4 mL per sample, including all sample preparations and system washes.

## 3. Potential clinical applications

Although glucose monitors and other enzyme-based systems still dominate the clinical biosensor market, the number of affinity-based sensors with potential for point of care (POC) use is rapidly increasing. Rapid immunosensors have been developed for detection of disease markers, such as prostate-specific antigen [15-17], C-reactive protein [17-19], and D-dimer [20, 21], among others [22-24]. Other clinical uses for immunosensors include detection of auto-antibodies present in patients with cancer [25-27] or autoimmune diseases [28-30], and testing of allergenic determinants [31]. A limited number of immunosensors has additionally been used to investigate the efficacy of vaccines or vaccination protocols [32], perform post-infection/post-intoxication serodiagnosis [33-38], or determine the immunologic responses to therapy [39].

The NRL Array Biosensor has also been used to determine “immunological signatures” of different human sera [40] using toxin antigens (tetanus toxin, SEB, diphtheria toxin) covalently immobilized in patterns on array substrates. Assays were first developed using animal antibodies and sera from commercial sources, using labeled tracers directed against IgG from the appropriate source. During the course of this development, a significant inhibition of antigen-antibody binding by high concentrations of serum was observed.

**Figure 4.**
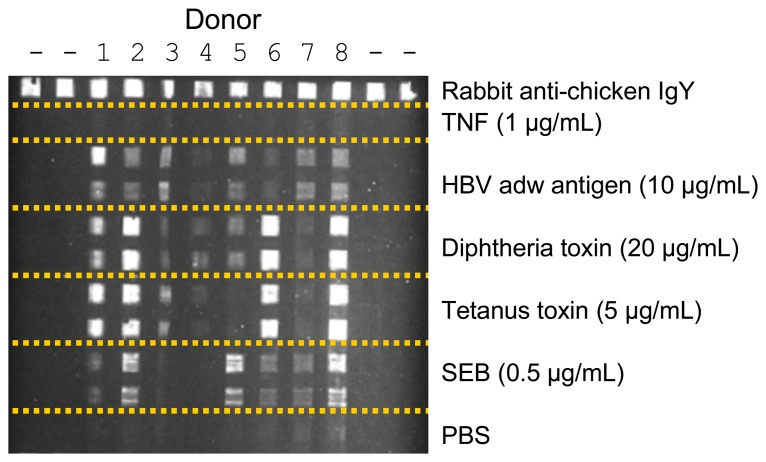
Pattern of binding by sera from 8 donors with varied immunologic histories. Arrays of immobilized toxins or antigens (shown to the right of image) were used to test sera of 8 donors (indicated above image) with varied histories of vaccination and exposure to antigens. After sera were incubated with the arrays, Cy5-labeled anti-human IgG was used as a tracer. TNF = human tumor necrosis factor [control], HBV adw = hepatitis B virus adw antigen [vaccine component], PBS = phosphate-buffered saline.

After the serum dilution was optimized for highest binding and signal generation, immunological profiles were determined for sera taken from eight volunteers with varied histories of vaccination and exposure to the antigens. Figure 4 shows a representative image of the antigen binding patterns from the eight individuals. As the times from vaccination or antigen/disease exposure to serum donation varied between the donors, the qualitative and quantitative differences could potentially be used to study the efficacy of vaccination, based on the levels of circulating antibodies. Since this study was published, Precision Photonics (www.precisionphotonics.com) has developed an inexpensive fully automated array biosensor for POC detection of disease markers, including circulating antibodies, with emphasis on applications in developing countries [4].

## 4. Expanding the capabilities - Use of alternative recognition species

The use of high affinity antibodies has allowed many immunosensors, including the NRL Array Biosensor, to reap the benefits of sensitivity, selectivity, and robustness in the presence of complex matrices. However, any system utilizing either antibody- and nucleic acid-based recognition requires some *a priori* knowledge of the suspect pathogen(s), as well as prior development of target-specific reagents; multiplexing of these target-specific reagents can become increasingly problematic when attempting to interrogate an unknown sample for large numbers of targets. In an effort to circumvent these limitations, other recognition elements are being explored as alternatives to complement or even replace conventional and engineered antibodies in detection assays. We have investigated the use of antimicrobial peptides and carbohydrates as two categories of alternative recognition species to expand the binding and detection capabilities of the NRL Array Biosensor.

### 4.1 Use of antimicrobial peptides for toxin detection

Antimicrobial peptides (AMPs) are produced by many organisms as part of their chemical defense system to protect them from pathogenic microorganisms. AMPs interact with target pathogens through invariant components of the cell membrane and exert their antimicrobial activity through membrane disruption, although some AMPs may have alternative mechanisms [41-43]. Their high affinities for bacterial membranes make AMPs promising candidates for use as effective recognition molecules in assays for bacterial species, and indeed, AMPs have proven quite effective in the detection and discrimination of multiple bacterial, viral, and rickettsial species [44-46].

**Figure 5.**
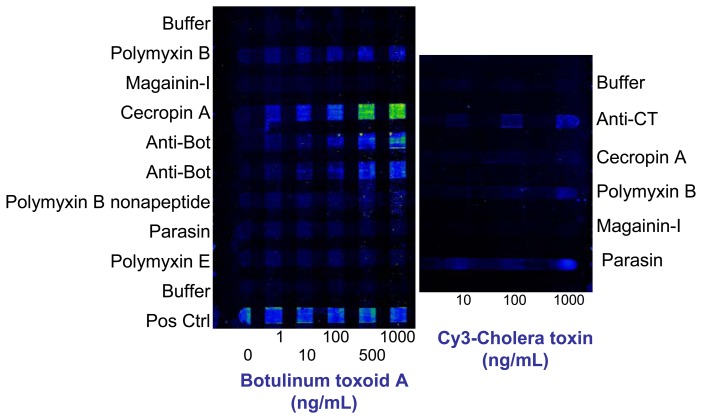
Detection of botulinum toxoid A and cholera toxin using antimicrobial peptides (AMPs). Immobilized AMPs were used as recognition species for capture of inactivated botulinum toxin A (left) and cholera toxin (right). After incubation of botulinum toxoid A with the arrays, Cy3-labeled tracer antibodies were used to interrogate the array for captured toxin (sandwich format). Cholera toxin assays utilized a direct assay format using Cy3-labeled cholera toxin (no tracer antibody).

Due to AMP's membrane-binding characteristics and their mechanism for cell killing, it is widely accepted that the natural targets of these AMPs are microbial cells. However, Garcia's group at Walter Reed observed that the AMP buforin-I could inhibit the protease activity of botulinum neurotoxin B in solution [47]. In large part because of Garcia's original observations and subsequent work [48, 49], we investigated whether other AMPs could be used to detect inactivated botulinum toxins A, B, and E as well as other toxins in assays analogous to the AMP-based bacterial assays [50]. As with the microbial assays, clear differences were observed in the patterns of binding between botulinum neurotoxoids A (Figure 5), B, and E (data not shown). In some cases, detection limits were improved when immobilized AMPs were used for target capture. “Direct” format assays and fluorescently labeled toxoid were required for detection of the E serotype toxoid, as the tracer antibody used in these studies did not bind to toxoid E. Intriguingly, Cy3-labeled cholera toxin was also observed to bind to several immobilized AMPs (Figure 5, right panel). These measurements will need to be repeated with a larger array of AMPs using a sandwich assay format to ensure that labeling of the toxin did not cause any artifacts. Results with the inactivated botulinum neurotoxins must also be repeated with live, active toxins under appropriate biosafety conditions.

### 4.2. Use of sugars as recognition elements for toxin detection

Binding of bacteria and toxins to various types of sugars (gangliosides and carbohydrates) has been documented in the literature [51-54]. Numerous research groups have exploited this natural phenomenon to design detection methods for pathogens [55-60]. We have successfully applied carbohydrates on the NRL Array Biosensor for detection of bacterial toxins [61-64]. The carbohydrates, the recognition elements of the sensor, were immobilized on the waveguide to form arrays that interrogated the test sample. Since the NRL Array Biosensor utilizes a glass substrate, the first step in immobilizing carbohydrates onto the surface of the waveguide was to attach a functional group onto the sugars. Using the functional group, the carbohydrate was then covalently tethered to the substrate. We employed three different immobilization methods to generate carbohydrate arrays on the NRL Array Biosensor.

#### 4.2.1 Immobilization using thiol-terminated linkers

In the first approach, two monosaccharides (GalNaC and Neu5Ac) and a trisaccharide (Neu5Acα2,3-Galβ1,4-Glc) were synthesized with a thiol-terminated linker (20 Å long) on the anomeric carbon and were immobilized onto the planar waveguide via a covalent bond between the sugars' thiol group and maleimide moieties on the glass [62, 63]. The tethered carbohydrate derivatives were exposed to various fluorescent labeled targets: cholera toxin (CT), tetanus toxin (TT), SEB, *Escherichia coli* O157:H7, *S. typhimurium* and *Listeria monocytogenes*. Of these targets, only Cy5-CT and Cy5-TT were found to bind to both GalNAc and Neu5Ac [62].

#### 4.2.2 Immobilization using amine-terminated linkers

The second approach for immobilization was to covalently attach amine-terminated carbohydrates onto the waveguide surface using the N-hydroxysuccinimidyl ester moiety of a bifunctional crosslinker. Binding of several toxins (CT, TT, ricin, botulinum toxoid A, and SEB), as well as bacterial species (*E. coli*, *S. typhimurium* and *Campylobacter*), to the immobilized carbohydrates was assessed in both sandwich and direct assay formats; mono-, di-, and trisaccharides were utilized as the immobilized capture species. Overall, only CT and Cy5-CT bound to any of the sugars; binding of these targets was limited to only two disaccharides, Galβ-1,4-Glcβ and Galβ-1,3-GalNAcβ (full dataset shown in Table 1, Supplemental Materials). Since the amine analogs of the thiol-sugars previously shown to bind CT or TT (β-GalNAc and α-Neu5Ac) did not show any binding in this study, the effect of the linking strategy was investigated. Using crosslinkers with identical *functional* moieties but different physical and chemical properties to immobilize the sugars, we found that binding of toxin to immobilized carbohydrates (but not to control antibodies) was greatly affected by the nature of the crosslinker [64], with no single variable (e.g., tether length or hydrophobicity) responsible for these differences. These results underscore that a “one-size-fits-all” approach may not be appropriate for immobilization of all small molecules.

#### 4.2.3 Immobilization using biotin-terminated linkers and multivalent scaffolds

A third strategy for immobilization of carbohydrate recognition molecules was also assessed – use of avidin-biotin interactions. NeutrAvidin-coated waveguides were used as sensor substrates, with biotinylated carbohydrates or carbohydrate-decorated scaffolds immobilized in arrays. Immobilized univalent biotinylated sugars (GlycoTech Corp., Gaithersburg, MD) possessing a short methyl linker between the sugar and the biotin did not bind to any of the targets tested (CT, TT, ricin). This lack of binding could be attributed to either steric hindrance caused by the large NeutrAvidin molecules or density issues. To mitigate both potential problems, multivalent polymers decorated with sugars were also investigated. These polymers consisted of a polyacrylamide (PAA) chain conjugated to biotin and sugars at varying stoichoimetries; a short linker connected each carbohydrate's anomeric carbon and the polymer backbone. These polymers essentially extend the distance between the biotin and sugar molecules, thus minimizing the potential for steric hindrance while also increasing the effective density of the carbohydrates.

A total of 22 sugar-polymers were screened for binding interactions to various toxin targets (Table 2 in Supplemental materials) using both direct and sandwich format assays. No significant binding was observed in assays for tetanus toxin, ricin, SEB, or botulinum toxoids B and E. Likewise, none of the bacterial cells tested (*E. coli*, *S. typhimurium*, *Campylobacter* and *Listeria*) had any appreciable interactions with the sugars. However, CT and botulinum toxoid A showed measurable binding interactions to a single monosaccharide (CT only - β-D-Gal), and to several disaccharides; no binding was observed to the three trisaccharides tested. No overlap in binding patterns was noted between the two toxins.

In general, the toxin-sugar recognition events using the multivalent scaffolds were not predictable based on the previous studies with the toxins and carbohydrates immobilized using thiol- or amine- terminated linkers (Sections 4.2.1, 4.2.2). Sugars previously shown to bind CT when immobilized using thiol-terminated linkers (β-GalNAc, α-Neu5Ac) did not interact with CT when conjugated to the multivalent scaffold. Conversely, Galβ-1,4-Glcβ – shown to bind CT but not botulinum toxoid A when immobilized via an amine-terminated linker – bound botulinum toxoid A but not CT when present on the multivalent scaffold. In only one instance did the binding pattern observed with the multivalent scaffolds match that previously observed with monovalent sugars: Galβ-1,3-GalNAcβ bound to CT and Cy5-CT, but not to any of the other toxins tested. Although the effects of the various immobilization strategies (i.e., via amine-, thiol-, and biotin-terminated linkers and scaffolds) are additionally confounded by linker effects, the use of various immobilized carbohydrates shows some promise for recognition of toxins with lectin-like activity. However, a significant effort must be placed on the systematic determination of the effects of linking strategies, tether effects, density/valency issues, stereochemistry and anomeric linkages of the sugars, and finally, signal generation (labeled toxin or tracer antibody) before these arrays can be implemented in a practical assay.

## Conclusion

The past five years have seen the evolution of the NRL Array Biosensor from a manual system requiring significant user intervention to a fully automated device with expanded capabilities. The utility of the automated Array Biosensor has been demonstrated in assays for toxins ranging from the very large (inactivated botulinum toxins) to intermediate-sized (cholera toxin, SEB) to those with low molecular weights (mycotoxins). Each target has been detected in a variety of different matrices including foods and environmental samples. Several blind trials were used to assess the true sensitivity, reliability, and logistical requirements of the system during real-world operation. This Array Biosensor has also been used for determination of immune antibody titres, with clear distinctions made between individuals of different immunologic histories. Finally, the versatility of this system to be used with multiple types of recognition schemes has been demonstrated.
